# Alternative Splicing in Breast Cancer and the Potential Development of Therapeutic Tools

**DOI:** 10.3390/genes8100217

**Published:** 2017-10-05

**Authors:** Nancy Martínez-Montiel, Maricruz Anaya-Ruiz, Martín Pérez-Santos, Rebeca D. Martínez-Contreras

**Affiliations:** 1 Centro de Investigaciones en Ciencias Microbiológicas, Instituto de Ciencias, Benemérita Universidad Autónoma de Puebla, Puebla CP 72570, Mexico; nancy.martinez.montiel@usherbrooke.ca; 2 Centro de Investigación Biomédica de Oriente (CIBIOR), Instituto Mexicano del Seguro Social (IMSS), Metepec, Puebla CP 74360, Mexico; manaya19@yahoo.com.mx; 3 Centro Universitario de Vinculación, Dirección de Innovación y Transferencia de Conocimiento, Benemérita Universidad Autónoma de Puebla, Puebla CP 72570, Mexico; martin.perez@correo.buap.mx; 4 Edificio IC11, Ciudad Universitaria, Col. San Manuel, Puebla CP 72570, Mexico

**Keywords:** breast cancer, alternative splicing, prognosis, therapeutics, expression

## Abstract

Alternative splicing is a key molecular mechanism now considered as a hallmark of cancer that has been associated with the expression of distinct isoforms during the onset and progression of the disease. The leading cause of cancer-related deaths in women worldwide is breast cancer, and even when the role of alternative splicing in this type of cancer has been established, the function of this mechanism in breast cancer biology is not completely decoded. In order to gain a comprehensive view of the role of alternative splicing in breast cancer biology and development, we summarize here recent findings regarding alternative splicing events that have been well documented for breast cancer evolution, considering its prognostic and therapeutic value. Moreover, we analyze how the response to endocrine and chemical therapies could be affected due to alternative splicing and differential expression of variant isoforms. With all this knowledge, it becomes clear that targeting alternative splicing represents an innovative approach for breast cancer therapeutics and the information derived from current studies could guide clinical decisions with a direct impact in the clinical advances for breast cancer patients nowadays.

## 1. Introduction

Breast cancer (BrCa) is now the most frequently diagnosed cancer and the leading global cause of cancer death in women, accounting for 1.38 million of cancer diagnoses and 458,000 casualties each year, being the most common type of cancer in women [1,2,3,4,5]. The total burden of BrCa doubled by the end of the last century and is likely to double by 2025. Although BrCa has a remarkably higher incidence in developed countries, half of the new cases and 60% of deaths are now thought to occur in the developing world. The incidence and mortality rate in these countries are even higher because of limited medical infrastructure and awareness [6,7]. Demographic studies on BrCa have revealed that the highest incidence is found in Western and Northern Europe, Australia, New Zealand and North America, occurring at ages between 40 and 50 in Asian countries and between 60 and 70 in Western countries [6,7,8,9,10]. The higher rates of BrCa in developed countries could be partially due to common lifestyle and reproductive factors [11]. Although with a lower reported incidence, BrCa remains the most common cause of cancer mortality for women also in developing countries. Even when the estimated difference could be related to incomplete reports from these regions [12], the “westernization” of developing countries may begin to resemble those in more developed countries, leading to an overall increase in the incidence of BrCa [13].

It is currently known that BrCa is a heterogeneous disease that comprises multiple subgroups with particular features including molecular variations, cellular background, sensibility to different treatments, clinical outcome and prognosis. Molecular profiling studies have identified five subtypes of BrCa according to the expression of estrogen receptor (ER), progesterone receptor (PR), and HER2/neu (HER2). According to these features, BrCa could be considered as luminal A (ER/PR+, HER2, luminal B (ER/PR+, HER2+), HER2 type (ER−/PR−, HER2+), TNBC or triple negative (ER−/PR−/HER2−) and normal types [14,15,16]. Critical differences between these subtypes are well established [17,18,19] and available models allow performance of in vivo studies [20]. Patient outcome of these basic intrinsic BrCa subtypes has been generally established [21] with median overall survivals of approximately 12, 20 and 56 months, respectively for patients with TNBC, Luminal and HER2 types [22,23,24].

## 2. Current BrCa Therapeutics

The treatment of BrCa has improved over recent years leading to an increased survival rate for patients through the application of several types of neoadjuvant and adjuvant therapies [25]. However, the application of personalized treatments could greatly improve opportunities for success in more patients. In general, neoadjuvant chemotherapy shrinks the tumor and is often used to avoid less extensive surgery [26,27] or to treat cancers that are too big to be removed at the time of diagnosis [28,29]. On the other hand, adjuvant chemotherapy is adopted after surgery in an attempt to reduce the risk of BrCa reappearance [30,31,32,33]. According to the 2011 and 2013 St. Gallen guidelines [34], the decision on systemic adjuvant therapies should be based on the surrogate intrinsic phenotype determined by ER/PR, HER2 and Ki-67 assessment with the selective help of first generation genomic test when available. Unfortunately, current tests do not consider molecular events that regulate the expression of these genes that could influence in some cases the response to the treatment.

Current therapies for BrCa also comprise immunotherapy [35], gene therapy [36] and drug therapies [37]. Immunotherapy includes immunomodulators and the use of antibodies to induce the death of cancer cells through different pathways. Using gene therapy, cancer cell death could be induced in order to slow or revert tumor growth. This therapy could include the use of viral particles [38] with the ability to replicate in BrCa cells where they produce for example a single chain antibody against VEGF [39]. Non-viral carriers [40] include cationic, anionic or neutral nanoparticles attached to nucleic acids [41]. Finally, RNA interference-based methods [42] have successfully silenced genes like CCL2 [43] and VEGF-C [44]. It would be interesting to modify some of these methods to target particular splicing isoforms for a more specific outcome. Regarding chemotherapy, combinations that include cyclophosphamide/fluorouracil and one of the following: doxorubicin, methotrexate, epirubicin or tamoxifen are often used to treat early BrCa [45], while in advanced stages the approach usually consists of single chemo drugs. Still, some combinations, such as carboplatin or cisplatin plus gemcitabine [46], veliparib-carboplatin [47], palbociclib-fulvestrant [48], lapatinib-isothiocyanates [49] are commonly used to treat cases of advanced BrCa. However, the ability of some of these drugs to alter gene expression is not always considered. The effect of these treatments on BrCa biology needs to be further analyzed but it should be taken into consideration when selecting the appropriate treatment for BrCa patients.

## 3. Alternative Splicing in Cancer

Several molecular mechanisms are involved in the regulation of gene expression, including epigenetic modulation, microRNAs and alternative splicing. Splicing consists of the removal of introns during pre-mRNA maturation and a combination of sequence elements and cellular factors contribute to splicing regulation [50]. Diverse combinations of splicing events could generate different mature mRNAs that could in turn produce distinct protein products due to alternative splicing (AS). AS is the main source of protein diversity involved in 90% of human gene expression [51,52], which has recently become a hallmark for cancer [53] and the target for the development of new therapeutic molecules [54,55]. In the past few years, genomic information related to different types of cancer has been annotated in several databases, including The Cancer Genome Atlas (https://cancergenome.nih.gov). High-throughput analysis of the RNA-seq data annotated in this database established novel splicing signatures that differ according to a specific type of cancer or to a distinct histological origin [56], granting AS a great capability as a prognostic and therapeutic tool. Currently, more than 15,000 AS events have been associated to different aspects of cancer biology, including cell proliferation and invasion, apoptosis resistance and susceptibility to chemotherapeutic drugs. Although the detailed mechanism responsible for splicing regulation has been extensively studied [57], the full relationship between this process and the implications in cancer biology, prognosis and treatment remains to be elucidated. For example, several apoptotic genes are alternatively spliced, producing isoforms with different and often opposite effects, including transmembrane receptors (Fas, Fas ligand), adaptor molecules (Bcl-x, survivin), caspases and executors [58]. Moreover, deregulation of splicing catalytic factors themselves has been also linked to BrCa development [59]. This has been reported for example for SRSF1 and RBM47 [60,61]. Altogether, this evidence strongly supports the pivotal role for AS mis-regulation in cancer progression.

AS occurs in all eukaryotes, but the main barrier to perform comparative studies for AS events arises from the differences observed across species, where the proportion of genes that undergo AS and the number of differential events detected could vary [62,63,64]. This species-specific behavior makes difficult the use of knockout mice to assess the functional relevance of a splice variant, considering that the correspondent human gene could show a different AS pattern.

## 4. Alternative Splicing Events Associated to BrCa

**Figure 1:**
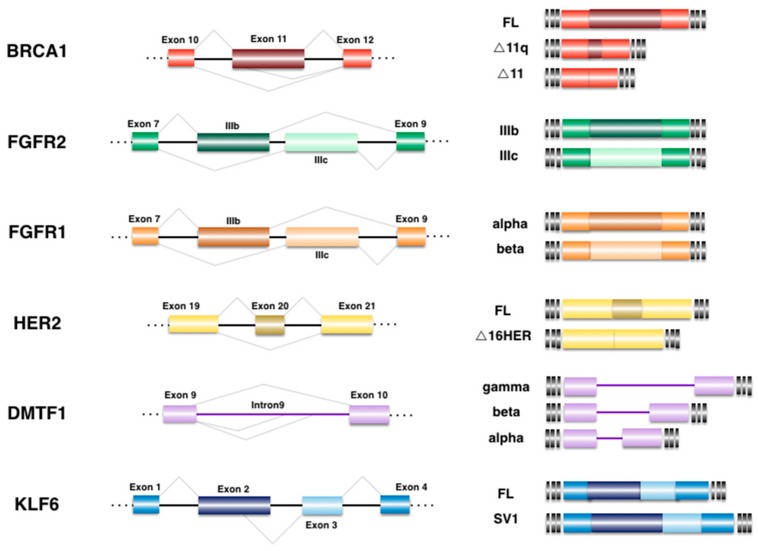
Alternative splicing events implicated in breast cancer (BrCa). Differential expression of various isoforms has been related to BrCa biology and tumorigenesis. The schematic representation of the pre-mRNA region that undergoes alternative splicing is illustrated at the left. The final outcome after alternative events is shown at right. Exons are depicted as boxes while introns are drawn as lines, alternative regions correspond to darker boxes, not drawn to scale. FL: full length product.

AS events that have been implicated in BrCa could in turn be considered as biomarkers or therapeutic targets for the early detection and treatment of the disease, as presented in this section and illustrated in Figure 1.

### 4.1. Breast Cancer 1 (BRCA1) 

This locus was defined more than 20 years ago as one of the major genes which mutations relates to a high risk to BrCa. *BRCA1* locates to the nucleus and is involved in DNA repair [65,66]. The nuclear localization signal of *BRCA1* lies in exon 11 and two isoforms generated through AS have been reported: *∆11*, which lacks the entire exon and *∆11q*, where most of exon 11 is missing. Both isoforms are cytoplasmic [67] and they seem to have tumor suppression activities [68]. Full-length *BRCA1* is down-regulated in BrCa tumors with an overexpression of the *∆11q* variant [69]. Recently, a comprehensive analysis of the annotation of *BRCA1* splice junctions identified 63 independent AS events in RNA samples from healthy individuals, with 10 predominant isoforms including *Δ11q*, plus 48 minor and 5 non-classifiable events [70], suggesting an intricate configuration of AS in this particular gene. As for biomarkers, alternative transcripts of the *BRCA1* gene in patients with BrCa and a family history of breast/ovarian cancer revealed the presence of three prevalent isoforms in blood samples that were probably pathogenic [71], which could be useful in evaluating cancer predisposition.

### 4.2. Cyclin D-Binding myb-like Transcription Factor 1 (DMTF1) 

In BrCa, *DMTF1* is overexpressed and has shown the ability to promote mammary tumorigenesis in a transgenic mouse model [72]. This gene encodes alternative isoforms with different functions in cancer [72,73]. Splicing variants of *DMTF1* include two isoforms shortened in the C-terminal domain designated as *DMTF1β* and *γ*, while the longer tumor suppressor isoform corresponds to *DMTF1α*. The short isoforms maintain a small portion of the myb-homology region and lack the DNA binding ability of the full-length protein. Regarding *DMTF1* expression in BrCa, alternative splicing occurred in about 30% of the samples analyzed, with relatively decreased *DMTF1α* and increased *DMTF1β* expression [74]. Moreover, information from the RNA-seq analyses performed by the ENCODE (Encyclopedia of DNA Elements) Consortium database showed an increase between 40 and 50% in the expression of *DMP1*β mRNA in human breast cancers, with slight variations depending on the histological origin. At the protein level, *DMP1β* is overexpressed approximately in 60% of tumor tissue in comparison to the surrounding normal tissue. It will be interesting to further explore if the overexpression of a given isoform is usually followed by a concomitant increase at the protein level for the alternative variant in the different BrCa-associated AS events.

### 4.3. Epidermal Growth Factor Receptor 2 (HER2) 

The *HER2* gene encodes an orphan receptor [75] with tyrosine kinase activity that is overexpressed in 30% of primary BrCa [76] usually correlating with enhanced tumor aggressiveness, lymph node metastasis and poor prognosis. The main isoform depicted for *HER2* due to AS corresponds to the *∆16HER* isoform, where a short stretch of 16 amino acids (residues 619–634) that conform exon 20 and code for the *HER2* extracellular domain is absent [77]. This deletion results in stable and active homodimer formation with enhanced activity and accelerated transformation [78,79].

*∆16HER2* is usually expressed in HER2+ BrCa, where it has been linked with resistance to trastuzumab (monoclonal antibody against *HER2*) in metastatic BrCa. For example, using transgenic mice, the expression of *∆16HER* accelerated mammary tumorigenesis and improved the response to trastuzumab [80]. A comparative analysis revealed that *∆16HER* activated the SRC pathway more effectively than *HER2*, while BrCa patients showing this genetic background received the greatest benefit from trastuzumab therapy [80]. Moreover, Wnt, Notch and epithelial–mesenchymal transition pathways related genes were activated in mammary tumor cell lines derived from *∆16HER* transgenic mice compared with full-length wild-type (WT) HER2+ cells [81]. Several studies have analyzed the expression of *∆16HER2* in relation to miR-7- and -15a/16-regulated signaling pathways involving BCL-2, EGFR, and/or SRC kinase [82,83,84] but the impact of *∆16HER2* on tumor pathology and therapeutic response in BrCa patients remains to be fully determined [85].

### 4.4. Fibroblast Growth Factor Receptor (FGFR) 

*FGFR1*, *FGFR2* and *FGFR3* are different isoforms generated through AS [86]. Increased expression of types 1 and 3 has been associated with poor overall survival in BrCa patients [87]. Two of the most studied variants include unique versions of domain III-immunoglobulin (Ig), termed *FGFR2-IIIb* and *FGFR2-IIIc* [86]. Ig domains are critical and regulate the affinity of *FGFR* binding to their ligands [88]. Another *FGFR* splicing event corresponds to the inclusion (*FGFR1-α*) or exclusion (*FGFR1-β*) of the first Ig domain and the linker region [89]. In this case, an increased expression of the β isoform with a decrease in the α isoform of *FGFR1* seems to correlate with reduced survival in BrCa patients [90].

### 4.5. Krüppel-like Zinc Finger Factor 6 (KLF6) 

*KLF6* has demonstrated tumor-suppressive abilities and the capacity to induce apoptosis; its functionality is often suppressed in cancer through somatic mutation or through alternative splicing [91]. The splice variant *KLF6-SV1* lacks three zinc-finger DNA binding domains depicted for the full-length protein, contains a novel C-terminal region and shows oncogenic properties, antagonizing directly the function of the full-length product [92]. It has been observed that BrCa tissues express high levels of *KLF6-SV1*, which correlates with multiple epithelial–mesenchymal transition markers analyzed in 294 primary breast tumors [93], suggesting a role for this variant in metastasis [94].

### 4.6. Survivin 

The gene *BIRC5* codes for survivin, a multifunctional protein involved in the control of apoptosis, angiogenesis and proliferation [95]. Survivin is overexpressed in a variety of human cancers and is considered a predictor of poor prognosis [96,97]. Besides the full-length transcript, six other splice variants have been identified for this gene: *survivin-2a*, *-2b*, *-2b+*, *-3b*, *-ΔΕx3* and survivin-image (*SI*), each correlating with tumor grade and size, cancer type, lymph nodes and estrogen receptors in BrCa with variable effect on patient prognosis [98]. For this reason, survivin and its splice variants have emerged as novel biomarkers for early diagnosis of BrCa in serum and tissue [99].

### 4.7. TP53 

*TP53* is a key tumor suppressor gene that induces apoptosis, commonly inactive in human cancer [100,101,102,103]. The human *TP53* gene produces multiple isoforms, which are differentially expressed in human breast tumors compared with normal breast tissue and correlate either with a positive (*β*/*γ* variants at the C-terminus) or negative impact (*Δ40*, *Δ133* at the N-terminus) on patients’ survival [104]. Isoforms truncated at the N-terminus lack one DNA-binding domain, while variants that differ at the C-terminus lack the tetramerization and C-terminal regulatory domains, which are replaced by unique amino acid sequences. These isoforms retain different features of TP53, suggesting that abnormal expression of the p53 isoforms may contribute to the loss of p53 tumor-suppressor activity in BrCa, indicating that several alternatively spliced genes could be involved in the same cell proliferation/survival pathways contributing to cell fate in different directions [105].

## 5. Prognostic Value of AS Variants in Breast Cancer

In the search for specific signatures for BrCa, several independent studies have recently characterized the mutational behavior of this disease [106,107,108,109]. The outcome of these studies has confirmed previously known cancer genes (e.g., *TP53* and *PIK3CA*) while they also report a long list of rarely mutated genes. The commonly used prognostic tools for BrCa evaluate the expression of different subsets of genes in RNA samples retrieved from the patients. These tools could analyze different numbers of genes (21 genes for Oncotype DX, 70 genes for MammaPrint, 97 genes for MapQuant). Depending on the tool, the information retrieved could help to classify tumors into different intrinsic subtypes in order to guide clinical decisions [110]. Unfortunately, for some cases the correlation applies only for a particular group or subtype of cancer and a complete list of biomarkers for BrCa should also evaluate AS events.

The AS of 600 cancer-associated genes was evaluated in a panel of 21 normal and 26 cancerous breast tissues [111] to identify independent markers for BrCa. This analysis revealed 41 specific markers for a ductal subtype and only two events had been previously associated to BrCa [112,113]. Moreover, five of these AS events were able to differentiate between tumor grades 1 and 3. In a few other studies, the AS profiles for BrCa has been analyzed, finding an overall agreement in isoform and gene expression levels in tumors, with specific differences in tumor subtypes and particular switching events [114,115]. All these applications are oriented towards a personal genomics approach that evaluates gene expression and the identification of AS events that appear particularly in BrCa.

## 6. Modification of Splicing Events as a Therapeutic Approach

Recently, very interesting procedures have been developed in order to reshape a particular splicing event. Briefly, the idea is to identify a splicing event that correlates with a precise oncogenic effect and change the splicing in the opposite direction towards the non-oncogenic activity. To accomplish this, the use of anti-sense oligos (ASO) or splicing-switching oligos (SSO) consisting of 15–20 nucleotides complementary to the mRNA are designed to recognize particular sequences involved in the regulation of the splicing event switching expression towards a particular isoform [116]. This approach is highly useful, specific and flexible, given its capability to artificially modify the expression of undesired splicing events, redirecting its expression towards a desired phenotype. In 2016, the Food and Drug Administration (FDA) approved the first drug designed according to the depicted approach, which is called SPINRAZA (nusinersen) and consists of an ASO designed to regulate the expression of the *SMN2* pre-mRNA towards the production of the full-length protein for the treatment of spinal muscular atrophy [117]. With the approval of this drug, an increase in the development of this type of therapeutic molecules is expected for different diseases, including cancer. Alternative splicing events depicted here are promising targets for this kind of therapy, particularly those isoforms that have been correlated with the more aggressive cases. Interestingly, targets could be more directed to BrCa like *HER2* or *BRCA1*, while some other genes could also be aimed also for some other types of cancer, such as *TP53*.

Several small molecules have been reported to alter the splicing mechanism as part of different drug screening efforts [118,119]. For example, it has been recently demonstrated that the antidiuretic amiloride has the ability to affect the splicing of several genes while showing anti-tumor activity [120]. Other small molecules with antitumor activity that block splicing correspond to a collection of microbial metabolites like spliceostatin or pladienolide [55] and even when the overall outcome for these molecules is the inhibition of cancer-related processes like proliferation and cell cycle, a complete inactivation of splicing could have adverse accessory effects for the organism.

## 7. AS events and Chemotherapeutic Response

The efficiency of cancer therapy is often affected by the appearance of resistant cancer cells due to different biochemical, pharmacological and molecular mechanisms [121]. Several studies suggest that the pharmacological regulation of AS can influence the response to different chemotherapeutic treatments [122] and it has been reported that AS events could be responsible for cell survival after chemotherapy due to the change in the expression of genes involved in apoptosis and drug metabolism. For example, it was shown that cisplatin induces changes in splicing events, which are mediated by the splicing factor SRSF4 and contribute to apoptosis in a process that involves class I PI3K [123]. In a different study, it was demonstrated that STF-083010, a novel drug that specifically blocks the splicing of XBP1, showed the ability to re-establish tamoxifen sensitivity to resistant MCF-7 cells [124].

Most commonly used anticancer agents trigger different apoptotic pathways and show variable changes in splicing. In this regard, etoposide, doxorubicin, camptothecin and derivatives improve the production of the pro-apoptotic isoform of caspase-2. However, the precise relationship between drug-resistance and AS needs to be further considered.

## 8. AS and Endocrine Therapy in BrCa

Like all types of cancers, BrCa has a multifactorial origin and it has been related to genetic background, hormone-associated reproductive factors, consumption of alcohol and type of diet, obesity, exposure to radiation, atypical hyperplasia of the mammary gland, older age at first birth and use of hormone therapy [98]. In fact, the use of oral contraceptives and hormone replacement therapy seems to contribute in some cases to the evolution of the disease, indicating that hormone signaling through both ER and PR receptors is also a factor in BrCa development [125,126]. Actually, ER and PR are important biomarkers for prognosis and response to therapy among patients with BrCa [127] and they are routinely measured in BrCa specimens. Approximately 40% of breast tumors are ER+/PR+ and these patients are most likely to respond to hormonal therapies, showing the best prognosis [125,128]. With this cellular background, therapy with selective PR and ER modulators is routinely applied in BrCa treatment [129,130]. Unfortunately, patients with advanced BrCa are usually PR−/ER− and become eventually unresponsive to selective PR and ER modulators [131,132], requiring chemotherapy as second-line treatment with severe adverse effects [133,134]. However, a small proportion of tumors is ER−/PR+ and still responds more favorably to hormonal therapies than ER−/PR− tumors [128,135]. This transition from a dependent to an independent status is a significant clinical problem, limiting the application of a less toxic endocrine therapy and advancing to a more aggressive phenotype for the disease [136,137].

Discrepancies between ER/PR status and response to hormone therapies could be due to the presence of AS variants of PR or ER that are either not detected or not distinguishable by current screening. The expression of different isoforms for these families of receptors adds another level of complexity to the response to endocrine therapy in BrCa. Alternatively, spliced genes involved in BrCa include several members of the steroid receptor superfamily with differing expression in normal and tumorigenic breast tissues [138,139]. Variant PR and ER expression in BrCa could provide a mechanism for abnormal proliferation, while the loss of normal expression for these receptors could inhibit normal response to the hormones, leading to discrepancies between the reported PR/ER status of a tumor and the progression of the disease or the response to endocrine therapies.

## 9. Conclusions

The exact splicing pattern associated with a particular BrCa type or stage still requires a broad characterization through molecular analysis of splicing isoforms in different patients. However, existing evidence strongly supports a pivotal role of alternative splicing on BrCa biology and innovative tools are under development to use splicing events with diagnostic and therapeutic purposes. In this regard, the information currently available could highly enrich a BrCa patient’s health history and hopefully, in the near future, splicing patterns will be analyzed on a regular basis in order to guide clinical decisions towards a personalized medicine.
